# The Impact of Cognitive Training on Cerebral White Matter in Community-Dwelling Elderly: One-Year Prospective Longitudinal Diffusion Tensor Imaging Study

**DOI:** 10.1038/srep33212

**Published:** 2016-09-15

**Authors:** Xinyi Cao, Ye Yao, Ting Li, Yan Cheng, Wei Feng, Yuan Shen, Qingwei Li, Lijuan Jiang, Wenyuan Wu, Jijun Wang, Jianhua Sheng, Jianfeng Feng, Chunbo Li

**Affiliations:** 1 Shanghai Key Laboratory of Psychotic Disorders, Shanghai Mental Health Centre, Shanghai Jiao Tong University School of Medicine, Shanghai, 200030, China; 2 Institute of Science and Technology for Brain-Inspired Intellegence, Fudan University, Shanghai, 200433, China; 3 Department of Computer Science, University of Warwick, Coventry CV4 7AL, UK; 4 Shanghai Changning Mental Health Center, Shanghai, 200335, China; 5 Department of Psychiatry, Tongji Hospital of Tongji University, Shanghai, 200065, China; 6 Department of Psychiatry, Tenth People’s Hospital of Tongji University, Shanghai, 200072, China; 7 Collaborative Innovation Center for Brain Science, Fudan University, Shanghai, 200433, China; 8 Shanghai Center for Mathematical Sciences, Shanghai, 200433, China; 9 Brain Science and Technology Research Center, Shanghai Jiao Tong University, Shanghai, 200030, China; 10 Bio-X Institutes, Key Laboratory for the Genetics of Developmental and Neuropsychiatric Disorders, Ministry of Education, Shanghai Jiao Tong University, Shanghai, 200030, China

## Abstract

It has been shown that cognitive training (CogTr) is effective and recuperative for older adults, and can be used to fight against cognitive decline. In this study, we investigated whether behavioural gains from CogTr would extend to white matter (WM) microstructure, and whether training-induced changes in WM integrity would be associated with improvements in cognitive function, using diffusion tensor imaging (DTI). 48 healthy community elderly were either assigned to multi-domain or single-domain CogTr groups to receive 24 sessions over 12 weeks, or to a control group. DTI was performed at both baseline and 12-month follow-up. Positive effects of multi-domain CogTr on long-term changes in DTI indices were found in posterior parietal WM. Participants in the multi-domain group showed a trend of long-term decrease in axial diffusivity (AD) without significant change in fractional anisotropy (FA), mean diffusivity (MD) or radial diffusivity (RD), while those in the control group displayed a significant FA decrease, and an increase in MD and RD. In addition, significant relationships between an improvement in processing speed and changes in RD, MD and AD were found in the multi-domain group. These findings support the hypothesis that plasticity of WM can be modified by CogTr, even in late adulthood.

Many studies have shown that cognitive training (CogTr) is effective for maintaining or improving cognitive function in elderly populations^1^. Single-domain CogTr, which targets specific cognitive domains such as memory, reasoning, processing speed, attention, strategy or problem-solving, could allow researchers to evaluate and elucidate regional or global effects related to the targeted domain. However, multi-domain CogTr, which involves collaboration and interaction with other mental processes^2^, is more useful in the real world and ideal for application with older adults.

The development of neuroimaging techniques has enabled non-invasive observations of functional and structural changes of brain plasticity *in vivo*. Although increasing age is accompanied by a decline in the volume of cortical and subcortical brain tissue^3^, some studies have demonstrated the plasticity of training-related increases in surface-based cortical thickness, grey matter volume and white matter integrity in various brain regions in older adults^4^,^5^. While cortical thickness and grey matter volume reflect the amount and state of neurons, the levels of white integrity imply changes of myelination, axon morphological changes and intravoxel coherence of fibre orientation^6^. In recent years, diffusion tensor imaging (DTI), as a magnetic resonance imaging (MRI) technique for analysing white matter, has enhanced the investigation of such microstructural properties in specific brain regions.

Fractional anisotropy (FA) and mean diffusivity (MD) are the two most commonly used indices to assess white matter integrity, as derived from DTI. While the former is a scalar value that refers to the coherence of the direction of water diffusion, the latter is the mean rate of free water diffusion across all three eigenvalues, representing the overall strength of water mobility in different regions of the brain^7^. The other two important indices, which refer to diffusivity parallel to and perpendicular to the axonal fibres, are axial diffusivity (AD) and radial diffusivity (RD) respectively. The former indicates axonal morphology, while the latter signifies the character of the myelin^8^,^9^.

Cross-sectional studies have identified that FA reductions, and MD and RD increases, are common in normal ageing^10^,^11^. However, changes in the above DTI indices, following cognitive intervention, have varied between regions in the literature of longitudinal studies to date^12^,^13^,^14^,^15^,^16^,^17^,^18^. Additionally, most studies have focused on short-term changes immediately after training, with only two reporting follow-up observations after a longer period. These latter studies were as follows. Lovden *et al*.^19^ discovered a trend of MD decreasing in the right hippocampal after four months of spatial navigation training, and returned to baseline four months after the completion of training. Meanwhile, Wolf *et al*.^20^ identified an FA increase in the corpus and genu of the corpus callosum; these improvements were maintained over a three-month follow-up period after the completion of a four-week logical reasoning training course. However, it remains unclear whether training-induced changes in DTI indices could be maintained over longer periods post-training (such as 6 or 12 months).

In the present study, we evaluated the long-term effects of CogTr on white matter microstructure using tract-based spatial statistics (TBSS)^21^.

Our first aim was to test whether long-term changes in white matter diffusivity were detectable across the groups at 12 months after training cessation. We hypothesised that if such changes were measurable, they would probably manifest as FA decreases, and/or MD increases, and/or RD increases, and/or AD decreases.

The second aim was to investigate whether CogTr could induce plasticity changes of white matter microstructure, and to further examine the differences between single-domain and multi-domain CogTr in this regard. Our previous study reported that both single- (focusing on reasoning) and multi-domain CogTr could improve cognitive ability and help maintain a functional state in community-dwelling older adults in China. In addition, compared with the control group, improved time-domain entropy in the cuneus and in the frontal areas, was detected in the single- and multi-domain CogTr groups, respectively^22^. Retained lateralisation of the left fronto-parietal network^23^ and enhanced resting-state functional connectivity within the default mode and central executive networks (CEN)^24^ was also observed after multi-domain CogTr across one year of follow-up. Therefore, we hypothesised that if changes in DTI indices were detectable after CogTr, they were likely to be manifested as increased FA and/or decreased MD and/or decreased RD in the frontal and parietal regions in the multi-domain CogTr group and in the occipital regions in the single-domain CogTr group.

The third aim was to assess whether training-induced changes in white matter integrity would be associated with improvements in cognitive function. Our previous studies found a correlation between training-derived improvement in global cognition and increased functional connectivity between the CEN network nodes in the multi-domain CogTr group^24^, as well as between delayed memory and time-domain entropy in the hippocampus in the single-domain group. On the basis of these findings, we hypothesised that training-related alterations in DTI indices would be correlated with improvements in cognitive performances.

## Results

**Figure 1:**
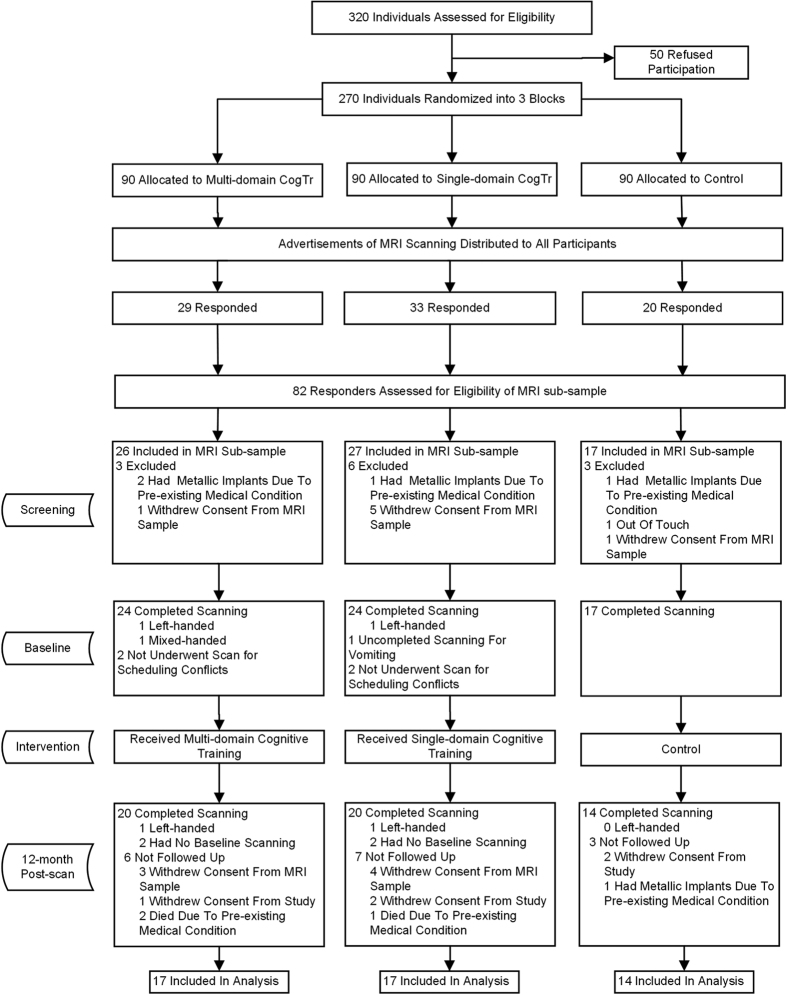
Flow chart of the participants’ recruitment. Adapted from previously reported study flow chart^22^ for excluding left-handed participants.

In all, 82 participants responded to our advertisement, representing 42.1% (82/195) of participants in the original trial^25^. After assessing eligibility for MRI contraindication and obtaining their informed consent, 70 participants were initially included in the MRI sub-sample; the remaining 12 were excluded due to having metallic implants, being out of touch or withdrawing their consent for the MRI scan. Out of our 70, four missed the baseline MRI scan due to scheduling conflicts, while one did not complete the scan because of nausea and vomiting. Between baseline and 12 months post-scan, seven participants withdrew their consent from the MRI sub-sample, five withdrew consent from the main trial (two had intestinal cancer, one had intracranial arteriovenous malformations, one was depressed following her husband’s death and one had difficulty with walking), three passed away and one did not undergo the MRI follow-up due to having metallic implants in hip arthroplasty (see study flow-chart in Fig. 1). Because differences related to handedness, as confounders in white matter, might have affected the results of the DTI indices^26^,^27^, two left-handed participants (one from each CogTr group) were excluded from the analysis. In the final reckoning, a total of 48 right-handed participants were used in this DTI analysis (17 in the multi-domain CogTr group, 17 in the single-domain group and 14 in the control group).

The baseline characteristics of these participants are described in Table 1. No differences in demographic features, medical history, cognitive performance or DTI indices were found across the three groups at baseline. One out of 17 (5.9%) participants in the single-domain CogTr group had hypertension, while this figure was zero in the other two groups (Fisher’s exact test, ***χ***^2^ = 1.750, two-tailed *P* = 1.000, n = 48). One out of 17 (5.9%) participants in the multi-domain group had diabetes mellitus; none of the other two groups’ members were so afflicted (Fisher’s exact test, ***χ***^2^ = 1.750, two-tailed *P* = 1.000, n = 48). All three groups included one participant with a history of heart disease (Fisher’s exact test, ***χ***^2^ = 0.470, two-tailed *P* = 1.000, n = 48). The medical histories of these subjects were all well-balanced.

### Longitudinal changes across groups

Significant (P < 0.05, family-wise error rate (FWE) corrected) longitudinal changes across groups (n = 48) were detected in all four DTI indices, with a bilateral distribution (see Supplementary Table S1 and Figure S1). FA decreases and RD increases were both interconnected in one cluster, with 65,814 and 50,747 voxels, respectively. The peak voxel of the FA cluster was in proximity to the posterior division of the right inferior temporal gyrus (33% probability) and temporal fusiform cortex (9% probability), and the anterior division of the right inferior temporal gyrus (9% probability), while that of the RD cluster was located in the right cingulum (hippocampus) (3% probability), close to the anterior division of the right temporal fusiform cortex (8% probability) and parahippocampal gyrus (5% probability).

MD increases were distributed in two clusters with 37,120 and 39 voxels. The peak voxel of the larger cluster was located in the left sagittal stratum, including the inferior longitudinal fasciculus and inferior fronto-occipital fasciculus, in proximity to the posterior division of the left temporal fusiform cortex (14% probability) and inferior temporal gyrus (7% probability). The smaller MD cluster was found to be nearby the left superior longitudinal fasciculus and corticospinal tract.

Both longitudinal AD decreases (14,295 voxels) and increases (12,111 voxels) were detected across the three groups over time. AD reductions were distributed in four clusters, where the peak voxel of the largest cluster (10,605 voxels, Cluster 4) was located in the left uncinate fasciculus (8% probability) and inferior fronto-occipital fasciculus (3% probability), in proximity to the left frontal orbital cortex (32% probability) and frontal pole (5% probability). The second largest cluster (2,861 voxels, Cluster 3) peaked at the right uncinate fasciculus (9% probability) and forceps minor (3% probability), while the two smaller clusters (732 voxels in Cluster 2 and 97 voxels in Cluster 1) peaked at the left inferior longitudinal fasciculus (42% probability) and forceps major (29% probability), respectively. AD increases were distributed in two clusters of similar size; the larger cluster (6,757 voxels, Cluster 2) peaked in the right tapetum, forceps major (13% probability) and right inferior fronto-occipital fasciculus (11% probability), while the smaller cluster (Cluster 1, 5,354 voxels) peaked in the left posterior thalamic radiation (including optic radiation), inferior fronto-occipital fasciculus (32% probability), inferior longitudinal fasciculus (24% probability), superior longitudinal fasciculus (temporal part) (18% probability) and superior longitudinal fasciculus (18% probability) in the left hemisphere.

A correlation analysis was performed to test the association between the age, sex, education and baseline values of the DTI indices. Age displayed a positive relationship (two-tailed Pearson’s *r* = 0.371, *P* = 0.009) with mean AD at baseline, even after controlling for sex and education (two-tailed partial *r* = 0.357, *P* = 0.015). No significant correlations between mean FA, MD or RD at baseline and age, sex and education were found.

### Training effect on cognitive performance

Cognitive measures at 12 months post-test can be seen in Supplementary Table S2. Table 2 shows the main effect of time, group and time × group interaction in terms of these measures. Only marginal time × group interactions (F (2, 45) = 2.872, *P* = 0.067) of the Repeatable Battery for the Assessment of Neuropsychological Status (RBANS)-delayed memory index score were detected. Table 3 lists Cohen’s *d* effect size of each group and net effect size (NES) of multi- and single-domain CogTr on each cognitive measure at 12 months post-test. The NES of multi-domain CogTr showed a small to medium effect of the training on the Chinese version of the Mini Mental State Examination (CMMSE), RBANS total score, language index score, attention index score, delayed memory index score, visual reasoning ability and Activity of Daily Living Scale (ADL) (NES = −0.779~0.613), while that of single-domain CogTr showed a similar impact on the RBANS total score, visuospatial/constructional index score, attention index score and visual reasoning ability (NES = 0.280~0.506). There was a transfer to untrained tasks after CogTr, including attention, in both training groups, while language and daily life functioning tasks were implemented in the multi-domain group only.

### Training effect on longitudinal changes of white matter

We hypothesised that cognitive training would have an influence on changes in DTI indices. First, a whole-brain voxel-wise analysis was performed to test for group differences in FA, MD, RD and AD at baseline; no differences among the three groups were detected. In addition, no group differences in changes of FA, MD, RD or AD between baseline and 12 months post-scan were found when controlling for the values of age, sex, years of education and baseline DTI indices.

**Figure 2:**
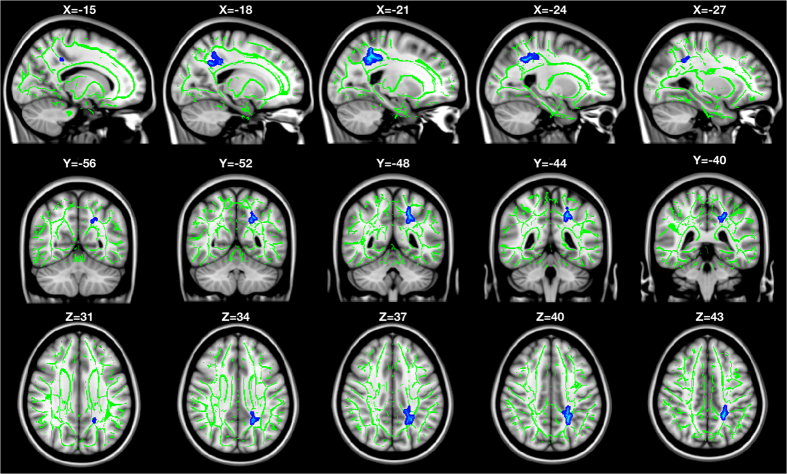
Cluster showing time × group interaction on RD between multi-domain CogTr group and control group. Based on a whole-brain voxel-wise comparison on RD at baseline and at 12 months post-scan using two-way mixed effect ANOVA for repeated measures. Significant (*P* < 0.025, FWE corrected) effects are inflated and displayed in blue to light blue, as they appear in the white matter skeleton shown in green. The background images are T1-weighted Montreal Neurological Institute (MNI) template brains denoted with MNI coordinates.

**Figure 3:**
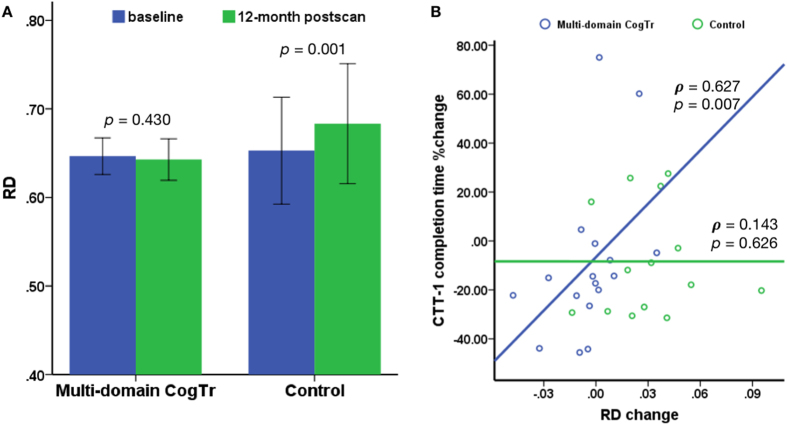
Impact of multi-domain CogTr on left posterior parietal white matter. (**A**) Bar plots with error bar illustrating mean RD (±SD) within the clusters in Fig. 2 at baseline (blue) and 12 months post-scan (green) for the multi-domain CogTr group and the controls. A significant (two-tailed *P* = 0.001) RD increase at 12 months post-scan from baseline was found in the controls, based on two-tailed paired sample *t* tests. (**B**) Scatter plot showing a positive relationship between RD changes within the cluster in Fig. 2 and percentage change of CTT-1 completion time in the multi-domain CogTr group two-tailed Spearman’s ρ = 0.627, P = 0.007, but not in controls. The blue and green circles and lines represent linear fit-lines for the multi-domain CogTr and control group, respectively.

As small to medium NES on cognitive measures were found in both intervention groups at 12 month post-test, a whole-brain voxel-wise analysis was conducted to examine further differences between each group using a two-way mixed-effect ANOVA. No significant time × group interactions in FA, MD, RD or AD were found between the multi- single-domain CogTr groups, nor between the single-domain and control group. However, a significant time × group interaction in RD was detected in the posterior regions of the brain (FWE corrected *P* < 0.05, distributed in four clusters, total cluster size = 2,404 voxels; see Supplementary Figure S2) between the multi-domain group and the controls. Due to the wide extent of the four clusters, a more conservative p threshold (*P* < 0.025, FWE corrected) was applied to detect more localised effects. This resulted in one parietal cluster in the left posterior corona radiata (14.4% probability) (cluster size = 396 voxels, MNI coordinates at peak voxel were [X = −18, Y = −52, Z = 34], corresponding to the left cingulum (cingulate gyrus) with 8% probability), in proximity to the left precuneus cortex and left superior parietal lobule (see Fig. 2). Association fibres, including the left cingulum (cingulate gyrus) (1.9% probability) and left superior longitudinal fasciculus (1.6% probability), went through the cluster. The mean RD value in this cluster was plotted for each group per time point; see Fig. 3A. Paired-sample *t* tests indicated a significant increase in RD in the controls (student’s *t* (13) = −4.250, two-tailed *P* = 0.001), while it remained stable in the multi-domain CogTr group (student’s *t* (16) = 0.809, two-tailed *P* = 0.430). An independent sample *t* test on the mean RD within this cluster indicated no significant group differences at baseline (student’s *t* (29) = −0.229, two-tailed *P* = 0.821).

Additionally, FA, MD and AD values within the cluster, showing a significant time × group interaction in RD, were examined. The mean FA for the multi-domain CogTr group was 0.350 mm^2^/s (SD = 0.025 mm^2^/s) at baseline and 0.355 mm^2^/s (SD = 0.027 mm^2^/s) at 12 months post-scan, respectively, while for the control group it was 0.352 mm^2^/s (SD = 0.053 mm^2^/s) at baseline and 0.330 mm^2^/s (SD = 0.057 mm^2^/s) at 12 months post-scan, respectively. A repeated measure ANOVA on FA revealed a significant time × group interaction (F (1, 29) = 19.221, *P* < 0.001), suggesting a greater FA reduction in controls. The same analysis revealed a significant main effect on time (F (1, 29) = 6.825, *P* = 0.014) without a similar effect on the group (F (1, 29) = 0.617, *P* = 0.439). Paired sample *t* tests revealed a significant decrease in FA in the controls (student’s *t* (13) = 4.999, two-tailed *P* < 0.001), whereas no change was found in the multi-domain CogTr group (student’s *t* (16) = −1.264, two-tailed *P* = 0.224). An independent sample *t* test on the mean FA within this cluster indicated no significant group differences at baseline (student’s *t* (29) = −0.136, two-tailed *P* = 0.893).

The mean MD within the cluster for the multi-domain group was 0.802 × 10^−3 ^mm^2^/s (SD = 0.043 × 10^−3 ^mm^2^/s) at baseline and 0.798 × 10^−3 ^mm^2^/s (SD = 0.051 × 10^−3 ^mm^2^/s) at 12 months post-scan, respectively, while for the control group it was 0.809 × 10^−3 ^mm^2^/s (SD = 0.091 × 10^−3 ^mm^2^/s) at baseline and 0.829 × 10^−3 ^mm^2^/s (SD = 0.103 × 10^−3^ mm^2^/s) at 12 month post-scan, respectively. A repeated measure ANOVA on MD disclosed a significant time × group interaction (F (1, 29) = 10.614, *P* = 0.003), suggesting a greater MD augmentation in the controls. No significant main effect on time (F (1, 29) = 3.898, *P* = 0.058) or on the group (F (1, 29) = 0.532, *P* = 0.472) was found. Paired sample *t* tests revealed a significant increase in MD in the controls (student’s *t* (13) = −3.017, two-tailed *P* = 0.010), whereas no change was identified in the multi-domain group (student’s *t* (16) = 1.143, two-tailed *P* = 0.270). An independent sample *t* test on mean MD within this cluster inferred no significant group differences at baseline (student’s *t* (29) = −0.281, two-tailed *P* = 0.781).

The mean AD within the cluster for the multi-domain group was 1.115 × 10^−3 ^mm^2^/s (SD = 0.065 × 10^−3 ^mm^2^/s) at baseline and 1.106 × 10^−3 ^mm^2^/s (SD = 0.072 × 10^−3 ^mm^2^/s) at 12 months post-scan, respectively; meanwhile, for the control group it was 1.121 × 10^−3 ^mm^2^/s (SD = 0.077 × 10^−3 ^mm^2^/s) at baseline and 1.124 × 0^−3 ^mm^2^/s (SD = 0.087 × 10^−3 ^mm^2^/s) at one year post-scan, respectively. No significant group differences in mean AD within this cluster at baseline (student’s *t* (29) = −0.255, two-tailed *P* = 0.801) were found upon implementation of an independent sample *t* test. We also pinpointed no time × group interaction (F (1, 29) = 1.827, *P = *0.187) or main effect on time (F (1, 29) = 0.354, *P* = 0.556) or on the group (F (1, 29) = 0.223, *P* = 0.640). Paired sample *t* tests revealed a marginally significant decrease in AD in the multi-domain CogTr group (student’s *t* (16) = 2.066, two-tailed *P* = 0.055); conversely, no significant alteration was seen in the controls (student’s *t* (13) = −0.401, two-tailed *P* = 0.695).

Time × group interaction in FA, MD or AD did not reach significance in a voxel-wise whole-brain analysis with a correction for multiple comparisons (*P *> 0.05, FWE corrected) between the multi-domain CogTr group and controls.

### Associations between cognitive functions and DTI changes

We explored the relationships between longitudinal DTI changes and percentage changes of cognitive measures by extracting the values of the DTI indices from the cluster showing significant time × group interaction in RD in the voxel-wise whole-brain analyses. As shown in Fig. 3B, in the multi-domain CogTr group, RD, MD and AD change correlated positively with the percentage change of CTT-1 completion time (RD: two-tailed Spearman’s ***ρ*** = 0.627, *P* = 0.007, n = 17; MD: two-tailed Spearman’s ***ρ*** = 0.588, *P* = 0.013, n = 17; AD: two-tailed Spearman’s ***ρ*** = 0.505, *P* = 0.039, n = 17). This indicates a greater RD, MD and AD decrease with a better improvement of CTT-1 completion time. No such relationship was found for FA (two-tailed Spearman’s ***ρ*** = −0.272, *P* = 0.291, n = 17).

No significant correlations between DTI changes and the percentage change of CTT-1 completion time were found for the control group (RD: two-tailed Spearman’s ***ρ*** = 0.143, *P* = 0.626, n = 14; MD: two-tailed Spearman’s ***ρ*** = 0.305, *P* = 0.288, n = 14; AD: two-tailed Spearman’s ***ρ*** = 0.486, *P = *0.078, n = 14; FA: two-tailed Spearman’s ***ρ*** = 0.015, *P* = 0.958, n = 14), implying that these associations were specific to the multi-domain group. Additionally, no significant correlations were detected between DTI changes and the percentage change of other cognitive measures in these two groups.

## Discussion

Our series of studies focusing on the effect of cognitive training intervention^25^ have demonstrated that it can maintain the improvement of memory, visual reasoning, visuospatial construction and attention for at least one year after course cessation in the community-dwelling elderly. Further functional connectivity analysis found that training-induced neural plasticity changes in frontal areas and the thalamus in the multi-domain CogTr group, and in the cuneus in the single-domain group^22^. There were three new findings in this particular study: (1) a significant long-term decrease in FA and increase in MD and RD were found at 12 months post-scan across the groups; (2) the parietal FA, MD, RD and AD were influenced by the multi-domain CogTr; and (3) a change in the parietal RD cluster, along with MD and AD changes within the cluster, was positively correlated with a percentage change of CTT-1 completion time, implying a relationship between behavioural gains and white matter microstructural alternations.

First, we investigated the impact of time on changes in DTI measurements. A paired t-test revealed a longitudinal FA reduction and MD and RD augmentation across the three groups, in line with previous cross-sectional^10^ and longitudinal studies^12^,^13^,^14^ on elderly populations. These changes may have resulted from the loss of small myelinated fibres, accumulation of water compartments in myelin sheaths or splitting and thickening of myelin lamellae due to advanced age^28^. The positive correlation between age and mean AD at baseline, and longitudinal AD increases across groups, were in keeping with two other longitudinal studies conducted on the elderly over two years^29^,^30^. In contrast to the AD decrease reflecting axonal injury, the AD increase was accompanied by a wavering FA and burgeoning RD, which perhaps indicates a normal ontological condition rather than a pathological process wrought by ageing^29^.

The next step was to investigate the long-term impact of changes to DTI indices. Significant time × group interaction in RD was detected in the multi-domain CogTr group, compared with controls, at 12-month post-test check. The latter also revealed a significant RD increase in the left posterior radiata, left cingulum of the cingulate gyrus and left superior longitudinal fasciculus in the control group, whereas no significant changes were detected in the multi-domain CogTr group. Further analyses revealed a significant FA decrease and corresponding MD increase within the cluster in the controls, which were absent in the multi-domain group, while a trend of AD decrease at 12 months post-scan was only detected in this group. The pattern of longitudinal FA or RD changes following cognitive training, which linked to the processes of demyelination and remyelination of white matter tract^9^, was similar to that seen in previously reported longitudinal studies on older adults^12^,^13^. The present findings augment other studies’ reports that myelination is a dynamic process, manifesting neurobiological and environmental interaction across one’s lifespan. It can be modified by experiences and affects information processing by regulating the velocity and synchrony of impulse conduction between distant cortical regions^4^,^31^, which is supported by experiments on cell cultures and animal and human studies^13^,^32^. FA enlargement and MD and RD reduction in the multi-domain CogTr group did not reach significance in the current study, which may be due to the relatively lower intensity and shorter duration^12^,^19^ (24 one-hour sessions over 12 weeks) or longer follow-up^12^,^16^,^17^,^19^,^20^ (12 months after training cessation).

In addition, we found a positive association, which was specific to the multi-domain CogTr group, between RD, MD and AD changes and percentage change of CTT-1 completion time, indicating the more RD, MD and AD fall, the greater are the benefits of perceptual tracking on sequencing and processing speed yielded by the multi-domain CogTr (more discussion about the training effect on cognitive performance see Supplementary Information). Decelerated processing speed is a hallmark of cognitive ageing and has a cascading effect on information processing, leading to decrements in working memory, inefficient inhibition of extraneous and task-irrelevant information in the environment and in memory, and deficits in sensory and motor information and attentional or processing resources^33^. The cluster showing training-related changes encompassed the posterior corona radiata, the cingulum of cingulate gyrus and the superior longitudinal fasciculus adjacent to the left precuneus cortex and superior parietal lobule.

The posterior corona radiata contains ascending fibres from the thalamus to the cerebral cortex and descending fibres from the fronto-parietal cortex to the subcortical nuclei and spinal cord; this is the neuroanatomical foundation of perceptual, motor, emotional and other higher cognitive functions^34^. White matter integrity of posterior corona radiata has previously been related to processing speed and episodic memory in lifespan studies^35^. Clinical studies have also reported that lower integrity in posterior corona radiata can cause impairments in processing speed in patients with type 1 diabetes^36^ and stroke^37^. The cingulum follows the entire course of the cingulate gyrus and contains long association fibres extending from the parolfactory area and the rostrum of the corpus callosum to the entorhinal area, whereas the superior longitudinal fasciculus connects the frontal and occipital lobes, with branching to the parietal and temporal regions. Diffusion studies have shown correlations between the integrity of the cingulum and superior longitudinal fasciculus with information processing speed, executive function and memory in both ageing and general patient populations^35^,^37^,^38^ (e.g. suffering from stroke, vascular cognitive impairment, Alzheimer’s disease or multiple sclerosis).

The adjacent grey matter includes the precuneus cortex and superior parietal lobule, which are key nodes of the default mode network^39^ and dorsal attention (frontal-parietal) network^40^, respectively. Two longitudinal studies into young adults^41^,^42^ revealed a significant grey matter volume (GMV) increase in the posterior parietal cortex after juggling training (a more complex visuo-motor training); one^41^ also detected FA increases in the white matter underlying the right posterior intraparietal sulcus. Functional imaging studies have also shown enhanced activation of the parietal cortex and precuneus after working memory training in young adults^43^. Reciprocal cortico-cortical connections between the precuneus cortex and superior parietal lobule may provide an anatomical basis for their functional coupling in visuospatial information processing and working memory^39^,^44^. In addition, recent studies have also revealed that the frontal-parietal/dorsal attention network, by engaging in top-down control of sensory processing, may serve as a basis for transfer between working memory tasks. The design of the multi-domain cognitive training in the present study was mainly based on visual materials involved with high-order cognitive functions (top-down)^40^ (such as face-name association memory, visuospatial map-reading and so on). Although the multi-domain CogTr group displayed a transfer to non-directly trained tasks of attention, language and daily life functioning, these behavioural gains did not statistically correlate with changes in parietal white matter integrity. A recent study focusing on training transfer in older adults^18^ found that far-reaching transfer to everyday problem solving from auditory perception training depended on increased white matter integrity in the ventral attention network (occipito-temporal and ventral fronto-thalamic pathways), which is thought to be engaged in bottom-up reorienting toward novel, behaviourally relevant stimuli. Taken together, top-down and bottom-up approaches may both form key components of effective intervention against ageing, and must be incorporated in future CogTr design.

In the present study, we found a trend of AD reduction without significant FA, MD or RD changes in the multi-domain CogTr group, and significant FA downgrading, MD and RD augmentation and no change in AD in the controls. The AD increase was interpreted as reduced axonal density or calibre-reflecting axonal injury (e.g. ischemic or chemically induced lesion or maldevelopment), whereas increasing axonal density or calibre-reduced interaxonal space was thought to have led to the AD decrease^45^. Increased or decreased AD, as reported in lifespan or intervention studies, depends on the specific tract assessed^10^,^11^. Strenziok *et al*.^18^ recently reported an AD boosting after auditory perception training in the occipito-temporal white matter, whereas AD was eroded in the same locale after working memory and strategic reasoning. Tang *et al*.^14^ also reported an AD downgrading in the left posterior corona radiata and left sagittal stratum after two weeks of meditation. Although inconsistent AD changes have been reported in the intervention literature, a positive correlation between AD reduction and CTT-1 completion time was found in the present study; this was specific to the multi-domain CogTr group, suggesting that an AD decrease may indicate enhanced white matter integrity (i.e. increasing axonal density or calibre) by the hand of multi-domain CogTr. Considering the complex relationships between AD changes, white matter microstructure and cognitive performance are still not fully understood; accordingly, the interpretation made here is advanced with caution, and more studies are required in order to increase our understanding.

Our analyses did not detect any significant differences of DTI indices in whole-brain analyses between the multi- and single-domain CogTr group, or between the single-domain group and the controls, which contrasts with previous findings claiming a distinct impact of single-domain CogTr on cognitive performance^25^ and neural functional connectivity, compared with the other two groups^22^. A recent study reported that RD decreased in the white matter connecting the frontal cortices, while MD diminished within the frontal and parietal lobes’ white matter after 100 hours of reasoning training in participants enrolled in a course to prepare for the Law School Admission Test^46^. Taken altogether, it is possible that such training merely induced regional functional changes in relation to behavioural gains, without structural alternation of white matter microstructure. Alternatively, the dosage of reasoning training in the current study was not strong enough to alter the structural connectivity of the brain, or the changes of white matter after this training did not exist long enough to be detectable at the 12-month post-scan follow-up.

One limitation of the present study is that the small sample size limited the statistical power to detect the time × group interactions among the three groups. Only the time × group interaction between the multi-domain CogTr and control groups was found. Second, although the training exposure and social contact were almost equal across the two intervention groups, the use of an active control or a social control group would be better for controlling the psychosocial and emotional factors of group-based intervention^47^. Third, the absence of short-term MRI scanning after the training sessions were completed led directly to a lack of short-term training-related changes, which may have helped us to further understand the underlying neural mechanism of multi/single-domain CogTr. Future studies, with sufficient statistical power and better design, are urgently required, in order to learn both the short- and long-term training effects on behavioural gain and neural plasticity changes.

In conclusion, our results demonstrate longitudinal changes in white microstructure, which differed between the multi-domain CogTr and control group over a 12-month follow-up period, and improvements in processing speed that correlated positively with RD reduction in the training group, indicating that multi-domain CogTr may have long-term protective effects against age-related changes to white matter degeneration. Additionally, DTI combined with other invasive neuroimaging (e.g. fMRI) techniques may be a powerful tool for facilitating the analysis of the underlying mechanism of CogTr and identifying core cognitive processes to develop specific training protocols for older adults, thereby improving maintenance and transfer.

## Methods

### Study design and participants

The present study was conducted using a subsample derived from our previous trial focusing on behavioural gains from cognitive training in healthy older adults, using the same methods of recruitment, eligibility criteria, randomisation, intervention and outcome assessment. The original trial^25^ employed a prospective, assessor-blind, block-randomised, controlled and parallel design. After assessing for eligibility, participants aged 65–75 were randomised into multi-domain CogTr, single-domain CogTr or control groups. The trial was approved by the Human Research Ethics Committee of Tongji Hospital, Tongji University (LL(H)-09-04) and prospectively registered with the Chinese Clinical Trial Registry (http://www.chictr.org.cn) (Registration Number: ChiCTR-TRC-08000732). All participants provided written informed consent. The methods were carried out in accordance with approved guidelines.

Eligible participants were older adults (65 ≤ age ≤ 75 years and educational level ≥1 year) without vision, hearing or communication difficulties, who were physically able to attend the training course for two sessions per week in Tongji Hospital. Participants were excluded for obvious cognitive decline, a diagnosis of Alzheimer’s Disease, serious functional decline (having difficulty with living independently), major neurological and/or psychiatric disorder (such as stroke, depression or schizophrenia) or cancer requiring current treatment. A further exclusion criterion was, having taken the CMMSE scoring <19 for elementary education and <24 for middle school and above^48^. After given their consent to participate in the trial and being randomised, all participants received an advertisement inviting them to participate in the neuroimaging subsample that was added to the main trial. Participants who responded were assessed for eligibility of MRI contraindication, and then signed a written MRI informed consent. Neither consent nor refusal to participate in the MRI sub-trial affected the procedures of the main study.

### Interventions

We developed cognitive training involving the multi- or single-cognitive domains, based on Gates and Valenzuela’s operational definition of CogTr, which includes four elements of repeated practice. We focused on tasks that require problem-solving, using standardised tasks and targeting specific cognitive domains^49^. The multi-domain CogTr comprised memory exercises (including verbal memory, episodic memory and face-name association memory), reasoning, problem-solving/strategy, visuospatial ability (map reading), handicrafts and physical exercise tips (stretching), whereas its single-domain counterpart was narrowly focused on reasoning training (including the “tower of Hanoi”, Raven’s progressive matrices, and numerical and verbal reasoning). A comprehensive description of these two interventions was reported in our previous paper^25^. In the present study, both intervention groups received a total quorum of 24 one-hour sessions over 12 weeks, while all three groups attended bimonthly lectures on healthy living.

### Cognitive measurement

The cognitive outcomes were changes across seven cognitive domains (memory, visuospatial/constructional, language, attention, executive functions, reasoning ability and global cognition) over four time points: at baseline and after 24 training sessions (immediately post-test), as well as 6 and 12 month after training cessation. Immediate memory (list learning and story memory), visuospatial/constructional (figure copy and line orientation), language (picture naming and semantic fluency), attention (digit span and coding) and delayed memory (list recall, list recognition, story memory and figure recall) index scores were obtained from the RBANS (Form A)^50^, which has been verified in terms of its good reliability and validity regarding the Chinese elderly community^51^. The Colour-Word Stroop test (CWST) was used to measure cognitive inhibition^52^ and the Colour Trials Test (CTT)^53^ was used to measure processing speed, sequencing and mental flexibility, and visual-motor skills^54^,^55^, while the visual reasoning test^56^ score reflected reasoning ability. The total scale index score of the RBANS and CMMSE scores represented global cognition.

### MRI data acquisitions

MRI scans were performed at baseline and 12 months after training cessation, using a Siemens 3.0 Tesla TIM-Trio scanner (Erlangen, Germany) at East China Normal University, Shanghai, China. For each participant, diffusion-weighted images were collected using an echo planar imaging (EPI) sequence with the following parameters: repetition time (TR)/echo time (TE) = 6732 ms/93 ms, flip angle = 90 degrees, FOV = 256 × 256 mm^2^, in-plane matrix size = 128 × 128, slice thickness = 2 mm without gaps, voxel size = 2.0 × 2.0 × 2.0 mm^3^, 50 axial slices. The protocol consisted of a non-diffusion T2-weighted acquisition (b-value = 0 s/mm^2^) followed by a 64-direction diffusion-weighted EPI scan (b-value = 1000 s/mm^2^), repeated twice in succession. The total scanning time was 16 minutes and 52 seconds. All acquisitions were examined for imaging artefacts. All MRI scans were reviewed by an experienced neuroradiologist to exclude significant injuries, signs of stroke or brain tumours, or other abnormalities. None of the participants were excluded based on radiological evaluation or poor MRI data.

### DTI analysis

All diffusion data was processed using the University of Oxford’s Center for Functional Magnetic Resonance Imaging of the Brain (FMRIB) Software Library (FSL) 5.0.8 (http://www.fmrib.ox.ac.uk/fsl/index.html)^57^. First, each participant’s data was pre-processed through an automated pipeline consisting of: (1) head motion and eddy current correction; (2) extraction of brain tissue from the b = 0 volumes using the Brain Extraction Tool (BET)^58^; (3) smoothing of the DTI image using the command “fslmaths” with a 1-voxel box kernel and the f-median flag^59^; (4) computation of the DTI scalar indices (FA, MD, AD and RD images). Next, to avoid removal of within-subject longitudinal differences, a nonbiased subject-specific template was used, as recommended by Engvig *et al*.^13^. Structural Image Evaluation, using Normalization, of Atrophy (SIENA)^60^ from FSL was run with option “-B”; the fractional intensity threshold parameter (f) was set to 0.2. After registering FA maps from baseline to 12 months post-scan (tp1 halfway registered maps) and vice versa(tp2 halfway registered maps), both volumes were resampled into the space at the halfway point between. Thereafter, a subject-specific template (base FA template) was created by averaging the two time points’ halfway registered FA maps. Next, all base FA templates were aligned into the Montreal Neurological Institute (MNI) template space using FMRIB’s nonlinear registration tool (FNIRT), and a mean FA map was created. Next, the mean FA map was thinned to create an average white matter tract skeleton, representing the centre of the tracts shared by all participants; the skeleton threshold was set at 0.2 as default to exclude grey matter and cerebral spinal fluid. Finally, each participant’s FA data from the base FA template was projected onto the mean skeleton, adopting the FA value from the local centre of the nearest relevant tract. After creation of the FA mean skeleton, the MD, AD and RD values for each participant were produced in a similar manner–halfway registration, warping and projecting the analogous data onto the mean FA skeleton. Changes of FA, MD, AD and RD maps from 12 months post-scan to baseline were calculated by tp1 halfway registered maps, subtracting from tp2 halfway registered maps using the command “fslmaths”.

### Statistical analyses

One-way analysis of variance (ANOVA), the nonparametric Kruskal-Wallis test and the Chi-squared test were performed to compare baseline characteristics between participants among the three groups, using IBM SPSS Statistics 22.0 (IBM Corporation, Somer, NY, USA). Independent sample *t*-tests, nonparametric Mann-Whitney tests and Chi-squared tests were conducted to compare characteristics between participants included in the final analysis and those who were excluded. The general linear model (GLM) repeated measure was used to investigate the training’s effect on the behavioural level. The model included the main effect for time and group and a time × group interaction term. Each cognitive measure was tested separately with covariates of the baseline RBANS total score.

Cohen’s d, as a measure of effect sizes of the *t* test for means^61^, was calculated using G*Power 3.1.9.2 (^©^ Franz Faul, Edgar Erdfelder, Albert-Georg Lang, and Axel Buchner, 2006, 2009). The NES was used to compare cognitive measures at 12 months post-test to baseline scores and control group scores. Bias-corrected NES of training was defined as: [(trained mean at 12-month post-test − trained mean at baseline) − (control mean at 12-month post-test − control mean at baseline)] ÷ pooled standard deviation at baseline, before applying a bias correction factor 1–3 ÷ [4 (n_T_ + n_c_ − 2) − 1]^62^. An operational definition of “small”, “medium” and “large” effect sizes were offered with d value equalling 0.2, 0.5 and 0.8, respectively^61^. Measures of percentage change (%change) in cognitive measures for each participant were derived by calculating the difference between variables at the two time points, then divided by variable at baseline [i.e. %change = (variable at 12 post-test − variable at baseline)/variable at baseline × 100].

Voxel-wise statistical analysis of the DTI data was carried out using the threshold-free cluster enhancement (TFCE)^63^ option in “randomise” in FSL by applying appropriate contrasts with GLM; *p* < 0.05, corrected for multiple comparisons (FWE), was considered significant. 5000 permutations were performed for each contrast and statistical inference.

First, we investigated the hypothesis that long-term changes of white matter diffusivity are detectable across groups as FA decreases, and/or MD increases, and/or RD increases and/or AD decreases. A whole-brain voxel-wise analysis was performed using two-sample paired *t* test on DTI indices between baseline and 12 months post-scan.

Second, we tested the hypothesis that the effect of cognitive intervention on white matter would last for as long as 12 months after training cessation. First, a whole-brain voxel-wise analysis testing for group differences in FA, MD, RD and AD at baseline was performed to exclude baseline differences. Next, a whole-brain voxel-wise F-test on changes of DTI indices among the three groups was conducted, controlling for age, sex, years of education and baseline DTI-indices values. A two-way mixed-effect ANOVA for repeated measures was conducted to further examine post hoc time × group interactions. The John Hopkins University (JHU) ICBM-DTI-81 White-Matter labels atlas^64^ and the JHU White Matter Tractography Atlas^65^ were used for anatomical labelling of regional differences of DTI indices from TBSS.

Finally, mean FA, MD, AD and RD values of all subjects were extracted from the brain regions, with significant group differences generated in the voxel-wise analysis, before being exported to SPSS using Pearson’s or two-tailed Spearman’s nonparametric correlation analysis according to data type. The aim was to test the relationships between change of DTI indices and age, sex, education and training-related changes in cognitive function.

## Additional Information

**How to cite this article**: Cao, X. *et al*. The Impact of Cognitive Training on Cerebral White Matter in Community-Dwelling Elderly: One-Year Prospective Longitudinal Diffusion Tensor Imaging Study. *Sci. Rep.* **6**, 33212; doi: 10.1038/srep33212 (2016).

## Supplementary Material

Supplementary Information

## Figures and Tables

**Table 1:** Demographic Characteristics and Cognitive Measures of Participants at Baseline.

	Multi-domain CogTr (n = 17)	Single-domain CogTr (n = 17)	Control (n = 14)	F/***χ***^2^	*P* (2-tailed)
Age, Mean ± SD (year)	71.1 ± 3.2	70.1 ± 4.1	69.1 ± 3.9	1.141	0.328
Male, n (%)	13 (76.5)	8 (47.1)	9 (64.3)	3.164	0.206
Education, years, Mean ± SD	10.9 ± 4.2	8.5 ± 4.6	11.4 ± 3.4	2.317	0.110
Retirement, n (%)	17 (100.0)	17 (100.0)	14 (100.0)	/	/
Married, n (%)	14 (82.4)	16 (94.1)	13 (92.9)	1.488	0.475
ADL, Mean ± SD^a^	14.0 ± 0.0	14.6 ± 2.4	14.1 ± 0.3	1.139	0.566
Cognitive measures (Mean ± SD)					
CMMSE (range 0–30)	27.8 ± 2.0	27.7 ± 1.6	28.4 ± 1.8	0.685	0.509
RBANS total score (range 0–160)	92.4 ± 16.2	91.6 ± 14.0	93.1 ± 15.0	0.037	0.963
Immediate memory (range 0–160)	85.1 ± 16.2	86.6 ± 16.1	84 ± 14.3	0.113	0.893
Visuospatial/Constructional (range 0–160)^a^	106.5 ± 13.2	99.6 ± 11.3	106.9 ± 19.1	2.007	0.367
Language (range 0–160)	91.9 ± 13.1	94.7 ± 10.2	93.3 ± 7.1	0.287	0.752
Attention (range 0–160)	91.2 ± 19.9	90.8 ± 15.6	91.5 ± 15.2	0.006	0.994
Delayed memory (range 0–160)	96.5 ± 17.8	97.6 ± 19.5	99 ± 13.1	0.083	0.921
CWST Colour interfere (second)	19.9 ± 14.3	17.4 ± 10.9	14.6 ± 7.6	0.820	0.447
CWST Word interfere (second)	38.6 ± 16.0	34.1 ± 12.1	39.7 ± 14.1	0.724	0.490
Visual Reasoning Test (range 0–9)^a^	5.8 ± 1.4	5.2 ± 2.1	5.9 ± 2.7	1.214	0.545
CTT-1 completion time (second)	80.8 ± 23.7	105.7 ± 59.0	87.2 ± 37.4	1.534	0.227
CTT-2 completion time (second)	160.4 ± 56.0	188.8 ± 116.0	153.5 ± 68.1	0.777	0.466
DTI indices (Mean ± SD)					
FA (mm^2^/s)^a^	0.365 ± 0.013	0.359 ± 0.017	0.355 ± 0.029	0.789	0.674
MD (10^−3^ mm^2^/s)	0.778 ± 0.021	0.784 ± 0.023	0.792 ± 0.038	0.959	0.391
RD (10^−3^ mm^2^/s)^a^	0.616 ± 0.023	0.625 ± 0.026	0.634 ± 0.046	1.331	0.514
AD (10^−3^ mm^2^/s)	1.102 ± 0.022	1.103 ± 0.019	1.108 ± 0.023	0.333	0.719

CogTr = Cognitive Training; ADL = Activity of Daily Living Scale; CMMSE = Chinese version of Mini Mental State Examination; RBANS = Repeatable Battery for the Assessment of Neuropsychological Status (Form A); CWST = Colour-Word Stroop test; CTT = Colour Trials Test; FA = fractional anisotropy; MD = mean diffusivity; RD = radial diffusivity; AD = axial diffusivity. CWST Colour interfere = Card C completion time–Card A completion time; CWST Word interfere = Card D completion time–Card B completion time.

^a^Kruskal-Wallis test for comparison.

**Table 2:** Cognitive training effect.

	Time	*P*	Group	*P*	Time × Group	*P*
CMMSE	1.586	0.214	0.340	0.714	0.292	0.748
RBANS total score	37.448	<0.001	0.213	0.809	1.539	0.226
Immediate memory	43.552	<0.001	0.034	0.967	0.117	0.889
Visuospatial/Constructional	0.217	0.643	0.349	0.707	1.523	0.229
Language	9.160	0.004	0.361	0.699	1.190	0.314
Attention	1.399	0.243	0.244	0.785	1.342	0.272
Delayed memory	47.557	<0.001	0.340	0.714	2.872	0.067
CWST Colour interfere	13.183	0.001	0.557	0.577	0.043	0.958
CWST Word interfere	0.966	0.331	0.628	0.538	0.044	0.957
Visual Reasoning Test	6.241	0.016	0.196	0.823	1.269	0.291
CTT-1 completion time	3.333	0.075	1.803	0.177	0.163	0.850
CTT-2 completion time	3.524	0.067	1.095	0.343	0.248	0.781
ADL	0.046	0.831	1.191	0.313	0.199	0.821

CMMSE = Chinese version of Mini Mental State Examination; RBANS = Repeatable Battery for the Assessment of Neuropsychological Status (Form A); CWST = Colour-Word Stroop test; CTT = Colour Trials Test; CWST Colour interfere = Card C completion time–Card A completion time; CWST Word interfere = Card D completion time–Card B completion time.

**Table 3:** Net effect of training group.

	Multi-domain CogTr		Single-domain CogTr		Control
	Cohen’s *d*	Bias-corrected NES	Cohen’s *d*	Bias-corrected NES	Cohen’s *d*
CMMSE	−0.084	0.234	−0.129	0.190	−0.312
RBANS total score	1.036	0.459	1.129	0.280	0.549
Immediate memory	0.805	−0.006	0.914	−0.155	1.475
Visuospatial/Constructional	−0.265	0.052	0.320	0.501	−0.210
Language	0.628	0.546	0.345	0.161	0.326
Attention	0.318	0.395	0.351	0.506	−0.187
Delayed memory	1.290	0.613	0.786	−0.039	0.896
CWST Colour interfere	0.371	−0.125	0.649	−0.011	0.709
CWST Word interfere	0.126	−0.012	0.201	0.123	0.102
Visual Reasoning Test	0.428	0.296	0.577	0.376	0.048
CTT-1 completion time	−0.247	0.093	−0.146	0.109	−0.504
CTT-2 completion time	−0.366	−0.145	−0.168	0.020	−0.332
ADL	^a^	−0.779	−0.042	−0.109	0.216

Cohen’s *d* effect size calculated from paired *t* test, positive value of CMMSE, RBANS, Visual reasoning and negative value of CWST, CTT and ADL for improvement at 12-month posttest; Bias-corrected NES comparing the means and standard deviations of training group and controls at 12-month posttest, positive value of CMMSE, RBANS, Visual reasoning and negative value of CWST, CTT and ADL is in favour of training group. ^a^Cannot be calculate for SD = 0; CMMSE = Chinese version of Mini Mental State Examination; RBANS = Repeatable Battery for the Assessment of Neuropsychological Status (Form A); CWST = Colour-Word Stroop test; CTT = Colour Trials Test; CWST Colour interfere = Card C completion time–Card A completion time; CWST Word interfere = Card D completion time–Card B completion time.
