# Supplementary material for: The Impact of Cognitive Training on Cerebral White Matter in Community-Dwelling Elderly: One-Year Prospective Longitudinal Diffusion Tensor Imaging Study
Source: Sci Rep. 2016 Sep 15;6:33212. doi: 10.1038/srep33212 (PMC5024122; doi:10.1038/srep33212)
Supplement: Supplementary Information [file srep33212-s1.pdf]

Supplementary Information

The Impact of Cognitive Training on Cerebral White Matter in Community-Dwelling Elderly: One-Year Prospective Longitudinal Diffusion Tensor Imaging Study

Xinyi Cao[1], Ye Yao[2,3], Ting Li[4], Yan Cheng[1], Wei Feng[5], Yuan Shen[6], Qingwei Li[5], Lijuan Jiang[1], Wenyuan Wu[5], Jijun Wang[1], Jianhua Sheng[1,*], Jianfeng Feng[2,3,7,8], Chunbo Li[1,9,10, *]

[1] Shanghai Key Laboratory of Psychotic Disorders, Shanghai Mental Health Center, Shanghai Jiao Tong University School of Medicine, Shanghai, 200030, China

[2] Institute of Science and Technology for Brain-Inspired Intellegence, Fudan University, Shanghai, 200433, China

[3] Department of Computer Science, University of Warwick, Coventry CV4 7AL, UK

[4] Shanghai Changning Mental Health Center, Shanghai, 200335, China

[5] Department of Psychiatry, Tongji Hospital of Tongji University, Shanghai, 200065, China

[6] Department of Psychiatry, Tenth People's Hospital of Tongji University, Shanghai, 200072, China

[7] Collaborative Innovation Center for Brain Science, Fudan University, Shanghai, 200433, China

[8] Shanghai Center for Mathematical Sciences, Shanghai, 200433, China

[9] Brain Science and Technology Research Center, Shanghai Jiao Tong University, Shanghai, 200030, China

[10] Bio-X Institutes, Key Laboratory for the Genetics of Developmental and Neuropsychiatric Disorders, Ministry of Education, Shanghai Jiao Tong University, Shanghai, 200030, China

[+]these authors contributed equally to this work

* Correspondence and requests for materials should be addressed to C.L. (chunbo_li@163.com) or J.S. (shengjianhua66@163.com)

Xinyi Cao[1]

Email: rekixinyicao@163.com

Ye Yao[2,3]

Email: yyao@fudan.edu.cn

Ting Li[4]

Email: liting2319@163.com

Yan Cheng[1]

Email: xiaoyanzi2001505@163.com

Wei Feng[5]

Email: ffww06@163.com

Yuan Shen[6]

Email: kmshy@sina.com

Qingwei Li[5]

Email: lianocd@tongji.edu.cn

Lijuan Jiang[1]

Email: ljjiang2012@163.com

Wenyuan Wu[5]

Email: wuwy@tongji.edu.cn

Jijun Wang[1]

Email: jijunwang27@163.com

Jianfeng Feng[2, 3, 7, 8]

Email: jffeng@fudan.edu.cn

Figure Legends

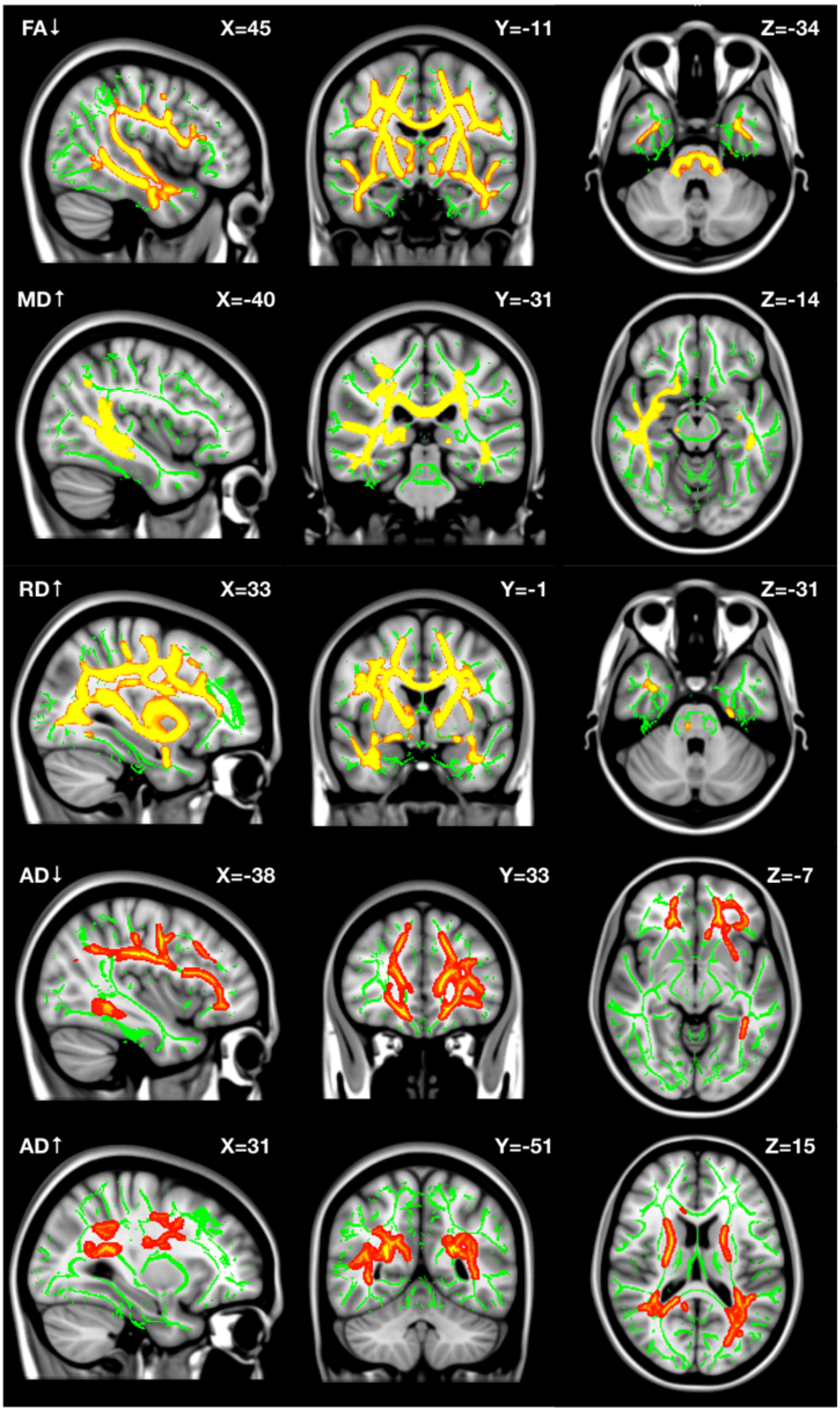

Supplementary Figure S1. Cluster showing longitudinal changes in DTI indices across group (n = 48). Based on whole-brain voxel-wise comparisons on

DTI indices at baseline and at 12-month post-scan using two-sample paired *t* test. Significant ($p < 0.05$, FWE corrected) effects are inflated and displayed in red-yellow as they appear in the white matter skeleton shown in green. The background images are T1-weighted Montreal Neurological Institute (MNI) template brain denoted with MNI coordinates.

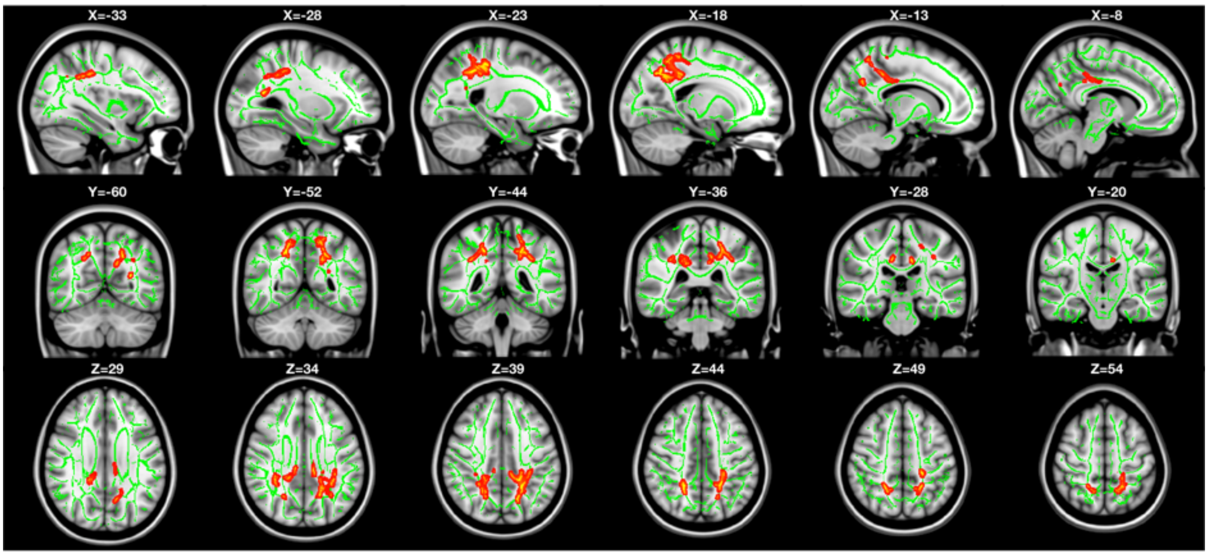

Supplementary Figure S2. Cluster showing time × group interaction on RD between multi-domain CogTr group and control group (n = 31). Based on a whole-brain voxel-wise comparison on RD at baseline and at 12-month post-scan using two-way mixed effect ANOVA for repeated measures. Significant (p <0.05, FWE corrected) effects are inflated and displayed in red-yellow as they appear in the white matter skeleton shown in green. The background images are T1-weighted Montreal Neurological Institute (MNI) template brain denoted with MNI Z-coordinates.

Tables

DTI indices (Change direction)	Cluster Index	Voxels	Peak 1-*P*	Peak MNI Coordinates		
				X	Y	Z
FA ↓	1	65814	1	45	-11	-34
MD ↑	1	39	0.952	-16	-28	-1
	2	37120	1	-40	-31	-14
RD↑	1	50747	1	33	-1	-31
AD↓	1	97	0.96	4	-37	13
	2	732	0.98	-46	-18	-18
	3	2861	0.988	14	38	-14
	4	10605	1	-38	33	-7
AD↑	1	5354	1	-36	-52	8
	2	6757	1	31	-51	15

Supplementary Table S1. Clusters of Significant (P < 0.05, FWE corrected) Longitudinal Changes in DTI Indices across Groups (n = 48). Based on whole-brain voxel-wise comparisons on DTI indices at baseline and at 12-month post-scan using two-sample paired *t* test. FA = fractional anisotropy; MD = mean diffusivity; RD = radial diffusivity; AD = axial diffusivity.

	Multi-domain CogTr (n=17)	Single-domain CogTr (n=17)	Control (n=14)
CMMSE (range 0-30)	27.7±2.2	27.5±2.3	27.9±2.3
RBANS total score (range 0-160)	106.2±12.9	102.2±16.8	99.6±13.7
Immediate memory (range 0-160)	101.5±22.9	100.7±18.8	100.5±14.3
Visuospatial/Constructional (range 0-160)	103.5±11.7	103.6±17.8	103.0±16.8
Language (range 0-160)	100.6±8.5	98.8±8.6	95.9±5.9
Attention (range 0-160)	95.9±15.1	96.3±18.2	88.9±15.2
Delayed memory (range 0-160)	117.6±12.2	108.1±19.4	110.2±14.8
CWST Colour interfere (second)	27.6±17.5	16.5±18.7	23.9±14.3
CWST Word interfere (second)	40.8±17.5	38.0±16.9	42.0±19.0
Visual Reasoning Test (range 0-9)	6.5±1.9	6.2±2.6	5.9±2.5
CTT-1 completion time (second)	73.5±35.0	101.2±64.7	77.1±29.2
CTT-2 completion time (second)	140.3±49.1	179.8±115.0	142.6±49.6
ADL	14.0±0.0	14.5±1.3	14.2±0.6

Supplementary Table S2. Cognitive Measures and ADL of participants included in analysis at 12-month post-test. CMMSE = Chinese version of Mini Mental State Examination; RBANS = Repeatable Battery for the Assessment of Neuropsychological Status (Form A); CWST = Colour-Word Stroop test; CTT = Colour Trials Test; CWST Colour interfere = Card C complete time – Card A complete time; CWST Word interfere = Card D complete time –Card B complete time. ADL = Activity of Daily Living Scale.

The baseline characteristics and cognitive performances of participants included in the final analysis and those excluded

There were no significant differences in age, sex, education, employment, marital status or baseline cognitive performances between the participants included in the final analysis and those excluded, except that those who were included in the final analysis displayed less word interference (Mann-Whitney U test, Z = -2.436, two-tailed *P* = 0.015, n = 70) than those who were excluded (see Table S3 and S4).

	Included in Analysis (n=48)	Lost to Follow-up (n=22)	t/χ^2	*P*(2-tailed)
Age, Mean± SD [a]	70.2±3.8	70.3±3.9	-0.175	0.862
Male, n(%)[b]	30(62.5)	13(59.1)	0.074	0.786
Education, years, Mean± SD [a]	10.2±4.2	10.0±3.5	0.181	0.857
Retirement, n(%)	48(100.0)	22(100.0)	/	/
Married, n(%)[c]	43(89.6)	18(94.7)[d]	/	0.667
ADL, Mean± SD [a]	14.2±1.4	14.0±0.0 [e]	0.668	0.506

Supplementary Table S3. Baseline Characteristics of the MRI-sample. [a] Based on Independent sample *t* test for comparison; [b] Based on Pearson Chi Square test for comparison; [c] Based on Fisher's Exact Test for comparison; [d] Data available for 19 participants lost to follow-up; [e] Data available for 18 participants lost to follow-up. ADL = Activity of Daily Living Scale.

	Included in Analysis (n=48)		Lost to Follow-up (n=22)		$t/Z/\chi^2$	*P*(2-tailed)
	Mean	SD	Mean	SD		
CMMSE (range 0-30)[a]	28.0	1.8	27.1	2.9	-0.905	0.365
RBANS total score (range 0-160) [b]	92.3	14.8	89.4	13.9	0.783	0.436
Immediate memory (range 0-160) [b]	85.3	15.3	84.5	13.3	0.226	0.822
Visuospatial/Constructional (range 0-160) [b]	104.2	14.7	98.7	15.9	1.398	0.167
Language (range 0-160) [b]	93.3	10.5	94.3	11.8	-0.343	0.733
Attention (range 0-160) [b]	91.2	16.8	85.4	16.7	1.341	0.184
Delayed memory (range 0-160) [b]	97.6	16.9	98.7	17.5	-0.245	0.807
CWST Colour interfere (second) [b]	17.4	11.5	17.4	11.1	0.025	0.980
CWST Word interfere (second) [a]	37.3	14.1	56.8	31.7	-2.436	0.015
Visual Reasoning Test (range 0-9) [b]	5.6	2.1	5.0	2.1	1.079	0.284
CTT-1 completion time (second) [a]	91.5	43.4	118.4	63.4	-1.917	0.055
CTT-2 completion time (second) [b]	168.4	84.7	204.0	89.8	-1.601	0.114

Supplementary Table S4. Cognitive Measures of the MRI-sample at Baseline. [a] Based on Mann-Whitney U test for comparison; [b] Based on Independent sample *t* test for comparison. CMMSE = Chinese version of Mini Mental State Examination; RBANS = Repeatable Battery for the Assessment of Neuropsychological Status (Form A); CWST = Colour-Word Stroop test; CTT = Colour Trials Test; CWST Colour interfere = Card C complete time – Card A complete time; CWST Word interfere = Card D complete time –Card B complete time.

Associations between RD changes and percentage change of CTT-1 completion time after correction for multiple comparisons

Methods: Effects were considered significant at $P < 0.2$, after correction for multiple comparisons using the Benjamini-Hochberg procedure, controlling for a false discovery rate (FDR) = 0.2[1,2], while that was considered significant at $P < 0.05$ using Bonferroni procedure, controlling for a familywise error rate (FWE) = 0.05[1]. The correction was conducted in R version 3.3.0[1]

Results: After correction for multiple comparisons, the positive relationship between RD changes and percentage change of CTT-1 completion time in multi-domain CogTr group (two-tailed Spearman's ρ = 0.627, P = 0.007, n = 17) remained significant using Benjamini–Hochberg procedure (corrected P = 0.084, false discovery rate = 0.20), while that became marginal significant using Bonferroni procedure (corrected P=0.084, familywise error rate = 0.05).

Discussion about the training effect on cognitive performance

The long-term impacts of CogTr were explored as they pertained to changes in cognitive performances. Only a marginal time × group interaction was revealed in delayed memory, without a significant main effect on the intervention groups. Since significant time × group interactions were detected in global cognition, delayed memory and visual reasoning in our original published study[3], the absence of significant interactions here should be interpreted with caution, considering the limitation of statistical power. The NES analyses were mostly in line with the previous research[3], which demonstrated that multi- and single-domain CogTr helped healthy community-dwelling older adults to perform better in terms of global cognition and benefited them in varying ways in specific cognitive domains. Based on a series of studies validating that cognitive training effects are specific to the ability targeted, it is not surprising that memory benefited the most from multi-domain CogTr, while visual reasoning and related visuospatial/constructional ability were the faculties that were bolstered to the greatest degree by single-domain CogTr. However, it was interesting to find a transfer to daily life functioning after multi-domain CogTr. No effects from the training on daily functioning were detected at the two-year follow-up to the ACTIVE study[4], while all three single-domain CogTr groups focusing on memory, reasoning or speed of processing resulted in slowed decline in instrumental ADL at their ten-year follow-ups[5]. The earlier detection of transfer to daily functioning in this study supported the notion that CogTr, involving different cognitive domains, might facilitate complicated interactions among multiple mental processes[6], which is closer to the daily life of our real world. Other confounding factors, such as personal beliefs or self-efficacy, also need to be considered in future research. More comprehensive discussions of the behavioural improvements of the entire sample were reported in Cheng et al.'s article[3].

References

1. McDonald, J. H.University of Delaware. *Handbook of Biological Statistics*. (2009).
2. Feng, W. *et al.* APOE Genotype Affects Cognitive Training Response in Healthy Shanghai Community-Dwelling Elderly Individuals. *J. Alzheimers Dis.* **47,** 1035–1046 (2015).
3. Cheng, Y. *et al.* The effects of multi-domain versus single-domaincognitive training in non-demented older people:a randomized controlled trial. *BMC Medicine* **10,** 30 (2012).
4. Ball, K. *et al.* Effects of cognitive training interventions with older adults: a randomized controlled trial. *JAMA* **288,** 2271–2281 (2002).
5. Rebok, G. W. *et al.* Ten-year effects of the advanced cognitive training for independent and vital elderly cognitive training trial on cognition and everyday functioning in older adults. *Journal of the American Geriatrics Society* **62,** 16–24 (2014).
6. Eckroth-Bucher, M. & Siberski, J. Preserving Cognition Through an Integrated Cognitive Stimulation and Training Program. *Am J Alzheimers Dis Other Demen* **24,** 234–245 (2009).
